# Development and Validation of a Fall Detection System for Older Adults Who Use Wheelchairs

**DOI:** 10.1093/geroni/igaf122.243

**Published:** 2025-12-31

**Authors:** Laura Rice, David Peeler, Peter Presti, Andrea Tangonan

**Affiliations:** University of Illinois College of Applied Health Sciences, Urbana, Illinois, United States; Georgia Tech, Atlanta, Georgia, United States; Georgia Tech, Atlanta, Georgia, United States; University of Illinois Urbana-Champaign, Urbana, Illinois, United States

## Abstract

Falls are a concern for the 1 million older adults who use a wheelchairs or scooters (WC/S) in the US. ∼60% of the population are affected by falls. After a fall, people who use WC/S spend an average of 9 minutes (range: 1-45 min) on the floor and 80% require assistance to recover. Remaining on the ground for an extended period of time after a fall is associated with an increased risk of future injurious falls, long term care admissions, and death. Automated fall detection devices can provide timely assistance and minimize the consequences of a long lie. Although used widely in ambulatory populations, current automated fall detection devices do not accurately detect falls from WC/S. In response, our team developed a system to detect falls among older adults who use WC/S and increase awareness of fall circumstances. A custom algorithm was developed to detect falls with high accuracy (>99%). 20 older adults, 4 care partners, and 10 clinicians and researchers provided feedback on the design of the fall detection system. Older adults and care partners indicated a desire for such a device, preference for a watch form, ability to modify who is contacted in the event of a fall, and ability to cancel a false alarm. Clinicians and researchers emphasized the need for such a system to guide user-centered fall interventions and better connection with patients. This system has good potential to increase the safety and quality of life of older adults who use WC/S.

